# The Regulatory Role of LncRNAs in Modulating Autophagy and Drug Resistance in Non-Small-Cell Lung Cancer: Focus on Targeted Therapeutic Approaches

**DOI:** 10.3390/biom15070968

**Published:** 2025-07-05

**Authors:** Shuncai Dai, Yuxin Zhong, Jianfu Lu, Linjiang Song

**Affiliations:** 1 School of Medical and Life Sciences, Chengdu University of Traditional Chinese Medicine, Chengdu 611137, China; 15208351536@163.com; 2 Biological Science Research Center, Southwest University, Chongqing 400715, China; zyx175080@163.com; 3 School of Intelligent Medicine, Chengdu University of Traditional Chinese Medicine, Chengdu 611137, China; l17378971337@163.com

**Keywords:** long non-coding RNA (lncRNAs), non-small-cell lung cancer (NSCLC), autophagy, chemotherapy resistance, precision medicine, biomarkers, targeted treatment

## Abstract

Lung cancer remains one of the leading causes of death associated with cancer globally, with non-small cell lung cancer (NSCLC) accounting for 80–85% of all lung cancer cases. Despite its high prevalence, the underlying mechanisms of NSCLC have not been completely clarified, and current therapeutic strategies face significant limitations. Recent research has revealed the important role of long non-coding RNAs (lncRNAs) in NSCLC, particularly in regulating processes such as autophagy and drug resistance. LncRNAs are a class of non-coding RNA molecules, typically with transcript lengths exceeding 200 nucleotides, and have been the subject of extensive investigation in recent years. Their involvement in critical cellular processes has opened up new research avenues for precision medicine in NSCLC. This review aims to offer a comprehensive analysis of the mechanisms by which lncRNAs regulate autophagy and drug resistance in NSCLC, explore their potential clinical applications as diagnostic biomarkers and therapeutic targets, and provide both theoretical foundations and practical guidance to advance precision medicine in this area. By deepening our understanding of the role of lncRNAs in NSCLC, this article also highlights the promising potential of lncRNA-based approaches for the diagnosis and treatment of this disease.

## 1. Introduction

Non-small-cell lung cancer (NSCLC) is the most common subtype of lung cancer, representing approximately 80–85% of all lung cancer cases [1]. This subtype includes adenocarcinoma, squamous-cell carcinoma, and large-cell carcinoma [2]. Despite considerable advancements in both diagnostic and therapeutic methods, NSCLC remains a significant public health concern worldwide due to its high incidence and mortality rates [3]. The 5-year survival rate for NSCLC patients continues to remain below 20% [4,5]. Chemotherapy, which is a standard treatment for NSCLC, faces limitations in effectiveness primarily due to the widespread occurrence of drug resistance [6]. This resistance plays a central role in the refractory nature of the disease [7]. In addition to contributing to treatment failure, chemoresistance is also strongly correlated with tumor recurrence and metastasis. These factors underscore the critical need for the development of novel strategies to address resistance and improve patient outcomes [8].

Autophagy is an essential, highly conserved cellular degradation process that plays a pivotal role in both the initiation and progression of cancer by maintaining cellular homeostasis through the removal of damaged organelles and proteins [9]. This process involves intricate molecular mechanisms and a complex regulatory network, with its dysregulation being implicated in a variety of diseases, including cancer and neurodegenerative disorders [10,11]. Recent studies indicate that the activation of autophagy in NSCLC cells is closely linked to tumor progression and resistance to chemotherapy [12,13,14].

Long non-coding RNAs (lncRNAs), a category of non-coding RNAs that exceed 200 nucleotides in length, have gained significant attention due to their broad involvement in the regulation of gene expression [15]. These lncRNAs play a role in various processes, including chromatin modification, transcriptional regulation, mRNA splicing, and stability control, through interactions with DNA, RNA, and proteins [16]. Their tissue-specific and spatiotemporal expression patterns have made them a central focus of cancer research [17]. Studies have shown that lncRNAs have a profound effect on tumor cell proliferation, invasion, metabolic adaptation, and treatment responses in NSCLC, primarily by regulating autophagy and pathways related to drug resistance [18]. Consequently, investigating the regulatory mechanisms of lncRNAs in NSCLC autophagy and drug resistance could not only enhance our understanding of their molecular mechanisms but also offer new perspectives for the development of lncRNA-targeted anti-resistance therapies [19].

## 2. Overview of LncRNA

**Figure 1:**
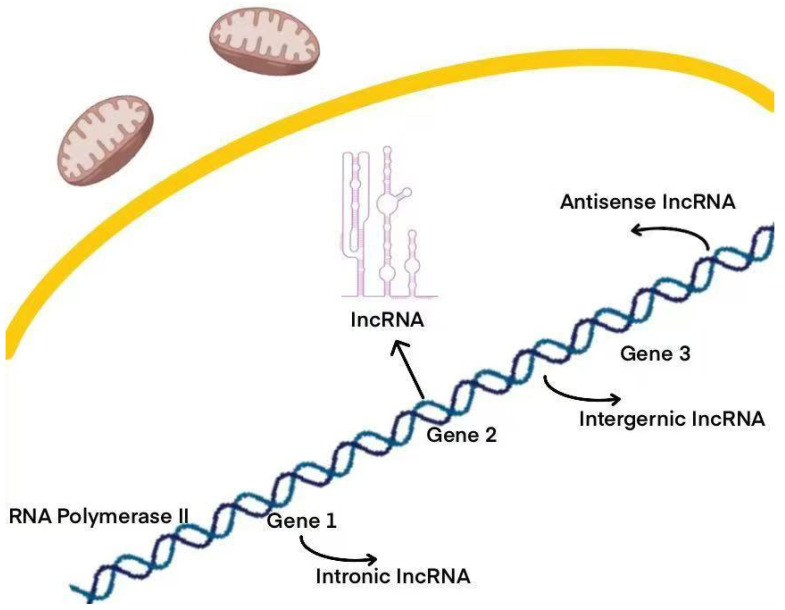
Various types of lncRNAs, such as intronic, intergenic, and antisense lncRNAs, are generated by genes. Each type plays a unique role in cellular functions and is essential for biological processes.

LncRNAs lack protein-coding potential and constitute a major portion of the human transcriptome [20]. These RNAs represent nearly 98% of the RNA transcribed from the human genome, which is non-coding. Interestingly, a significant portion—nearly three-quarters—of the genes within the less than 2% of the human genome that do encode proteins are actively transcribed into non-coding RNAs [17,21]. The origins of lncRNAs are varied; they can emerge from enhancer regions, independent promoters, or share promoters with both coding and non-coding genes [19]. This genomic diversity contributes to the wide range of functional and regulatory roles that lncRNAs play [22]. Consequently, lncRNAs are classified into eight distinct categories based on their genomic origins: intergenic lncRNAs, intronic lncRNAs, enhancer lncRNAs, promoter lncRNAs, natural antisense/sense lncRNAs, small nucleolar RNA-derived lncRNAs (sno-lncRNAs), bidirectional lncRNAs, and non-polyadenylated lncRNAs [23,24,25] (Figure 1). Each type plays a unique role in cellular functions and is essential for biological processes. Across mammals, all lncRNAs exhibit common structural, functional, or mechanistic characteristics, including transcription by RNA polymerase II, which is associated with a 5′ cap structure and, optionally, a 3′ polyA tail [26,27,28]. In addition, lncRNAs play a pivotal role in regulating various biological processes, such as gene expression, alternative splicing, chromatin modification, the modulation of protein activity, and post-transcriptional mechanisms [29]. These processes enable lncRNAs to directly or indirectly influence the onset and progression of cancer.

LncRNAs have the ability to localize in the nucleus, cytoplasm, or both compartments, and their functionality is largely influenced by their precise subcellular positioning [30]. The localization not only determines the mechanisms through which lncRNAs exert their functions within the cell but also plays a role in regulating the biological processes they contribute to. In the nucleus, lncRNAs are commonly involved in regulating gene expression, modifying chromatin, and controlling transcription, while in the cytoplasm, they mainly regulate processes such as protein synthesis, RNA stability, and translation [31].

In the nucleus, certain lncRNAs play a pivotal role, particularly in the formation and stability of nuclear speckles, sub-speckles, and chromatin structures [32,33]. By interacting with nuclear components, these lncRNAs help preserve the functionality of these crucial nuclear substructures [34]. Furthermore, another group of nuclear lncRNAs influences gene activation through epigenetic mechanisms by recruiting chromatin-modifying factors, thereby controlling the activity of specific loci [35]. These lncRNAs typically perform their roles by modifying chromatin and interacting with transcription factors, which in turn affect gene expression patterns [16].

Beyond the conventional nuclear lncRNAs, there are also other distinct forms, such as competing endogenous RNAs (ceRNAs) and circular RNAs, which accumulate over time within the cell and play vital roles [15]. These stable lncRNAs interact with miRNAs, serving as “decoys” or “sponges”, thus indirectly modulating gene expression and influencing the post-transcriptional regulation of downstream target genes [36]. In addition, these lncRNAs regulate cellular processes in the nucleus and affect translation and stability in the cytoplasm through the “sponge effect” of miRNAs [37].

Considerable progress has been made in the investigation of lncRNAs as cancer biomarkers, with these molecules influencing tumorigenesis, progression, metastasis, and resistance to chemotherapy through various mechanisms [38]. Numerous lncRNAs, including HOTAIR, MALAT1, and PVT1, are crucial in a wide range of cancers and have significant clinical relevance [39]. As a result, the detection of circulating lncRNAs in serum can aid in the early diagnosis of specific cancer types [40]. Furthermore, lncRNAs are also detectable in urine, where they function as biomarkers for conditions such as kidney transplant rejection [41] and as staging markers for bladder cancer [42]. In a noteworthy development, the U.S. Food and Drug Administration (FDA) recently approved PCAT-3 lncRNA as a biomarker for prostate cancer in urine, demonstrating higher sensitivity and specificity compared to the traditional prostate-specific antigen (PSA) blood test [42]. Additionally, lncRNAs can be identified in saliva, which makes it an important source for cancer biomarkers [43]. For example, certain lncRNAs, like HOTAIR, are present in saliva, and their expression levels correlate closely with high expression in metastatic tissues, thereby serving as reliable diagnostic markers for bladder cancer [44]. As our understanding of lncRNA functions expands, these molecules are poised to offer new avenues for precision and personalized cancer therapies.

## 3. LncRNAs in Non-Small-Cell Lung Cancer

**Figure 2:**
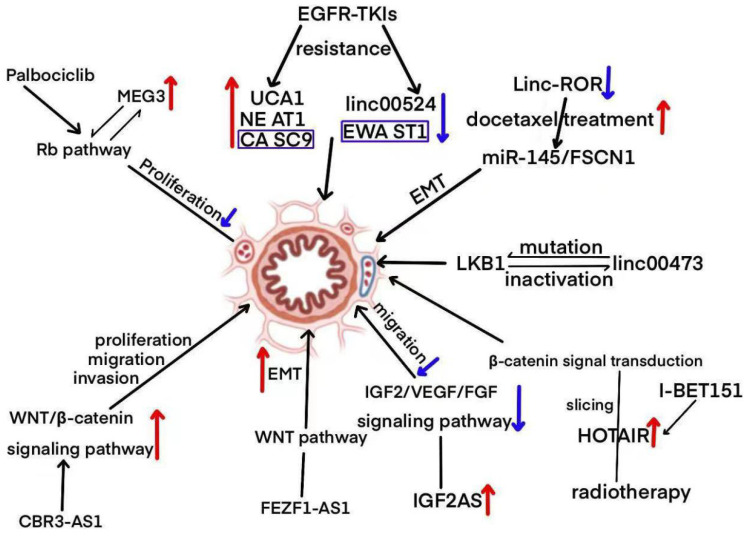
A diagrammatic representation depicting the role of lncRNAs in the regulation of NSCLC signaling pathways.

LncRNAs play a critical role in cellular development and differentiation, mainly by regulating gene expression and maintaining or modulating cellular homeostasis [45,46]. Abnormal lncRNA expression has been identified in a variety of cancer types, including those affecting the hematopoietic system, urinary system, lungs, stomach, breast, pancreas, and colorectal cancers [41,42,44,45,46,47]. As a result, alterations in these molecules are actively studied in non-small-cell lung cancer to identify potential clinical biomarkers for diagnosis, prognosis, and treatment [48,49]. Numerous lncRNAs are strongly associated with non-small-cell lung cancer, playing a significant role in clinical outcomes by regulating processes such as tumor migration, invasion, and other related activities [18] (Figure 2). As illustrated in Figure 2, these lncRNAs participate in diverse biological processes such as proliferation, epithelial–mesenchymal transition (EMT), migration, and drug resistance. For example, UCA1, NEAT1, and CASC9 are upregulated and contribute to EGFR-TKI resistance, while linc00524 and EWSAT1 are associated with reduced resistance. MEG3, regulated via the Rb pathway and enhanced by Palbociclib, inhibits cell proliferation. FEZF1-AS1 and CBR3-AS1 activate the WNT/β-catenin pathway, thereby promoting EMT and cellular invasion. Linc-ROR affects EMT and enhances docetaxel sensitivity through miR-145/FSCN1 signaling. IGF2AS upregulates the IGF2/VEGF/FGF signaling axis, driving cell migration. HOTAIR is implicated in radiotherapy resistance and β-catenin signaling, with its effects mitigated by I-BET151. Furthermore, LKB1 mutations, in association with linc00473 inactivation, facilitate EMT and tumor migration. Collectively, these findings underscore the multifaceted roles of lncRNAs in NSCLC pathogenesis and therapeutic resistance, reinforcing their potential as valuable targets for clinical intervention.

In non-small-cell lung cancer, lncRNAs play a vital role in both carcinogenic and tumor-suppressive pathways. These molecules regulate essential cellular processes such as proliferation [50], apoptosis [51], metastasis [52], and angiogenesis, which significantly influence the tumor microenvironment’s complexity [53,54]. The dysregulation of specific lncRNAs, including LINC01140, MALAT1, HOTAIR, and H19, is closely linked to tumor progression, poor prognosis, and resistance to treatment [55].

### 3.1. LINC01140

LINC01140, a long non-coding RNA situated on chromosome 1q41, plays a multifaceted role in NSCLC [56]. Research has shown that this RNA is overexpressed in both NSCLC tissues and cell lines, where it binds to several microRNAs, including miR-33a-5p, miR-33b-5p, miR-377-3p, and miR-155-5p [57]. This binding process alleviates the suppression of c-Myc and PD-L1, subsequently enhancing tumor cell proliferation, migration, invasion, and immune escape [58]. Furthermore, in vivo experiments demonstrate that silencing LINC01140 significantly inhibits tumor growth and amplifies the anti-tumor effect of cytokine-induced killer cells, highlighting its potential as an immunotherapy target [59]. Interestingly, in certain NSCLC cases, the expression of LINC01140 is downregulated [60]. In these cases, LINC01140 competitively binds to miR-4742-5p, regulating TACC1 expression, which suppresses both cell invasion and cisplatin resistance, exhibiting an anti-cancer effect [56]. Moreover, the expression level of LINC01140 has the potential to serve as a biomarker for both immunotherapy response and prognosis in NSCLC [61].

### 3.2. MALAT1

MALAT1 (Metastasis-Associated Lung Adenocarcinoma Transcript 1) is a nuclear lncRNA that is highly conserved and was initially explored for its involvement in the metastasis of NSCLC [62]. In patients with advanced lung adenocarcinoma, its expression level is reported to be 8.4 times higher compared to the normal group [63]. This lncRNA influences the expression of genes linked to metastasis, rather than RNA splicing, and plays a crucial role in promoting NSCLC cell migration as well as the formation of pulmonary tumor nodules [64]. Serving as a competing endogenous RNA (ceRNA), MALAT1 interacts with miR-206, miR-124, and miR-200a-3p, thereby activating the Akt/mTOR and STAT3 signaling pathways, which in turn facilitates EMT and cell invasion [65]. Moreover, by binding to miR-197-3p, MALAT1 upregulates STAT3 expression, thus enhancing the resistance of NSCLC to gemcitabine treatment [66]. Transcriptomic analysis further demonstrates that MALAT1 regulates a variety of cancer-related signaling pathways, underscoring its pivotal role as an oncogenic factor in NSCLC [67]. In addition, MALAT1 binds to miR-202, relieving the inhibition of MMP2/MMP9, which further supports the remodeling of the tumor microenvironment [68].

### 3.3. HOTAIR

HOTAIR (HOX Transcript Antisense RNA) is a lncRNA located within the HOXC gene cluster and is widely acknowledged for its oncogenic functions in various cancers [69]. In NSCLC, HOTAIR exhibits significant overexpression, and studies have shown that this overexpression is correlated with several factors, including lymph node metastasis, tumor lymph node involvement, distant metastasis, Duke staging, histological type, degree of differentiation, and a poor prognosis [70]. In NSCLC, HOTAIR negatively regulates the chemokine CCL22, thus inhibiting T cell immune activity in the tumor microenvironment, while promoting cell proliferation, migration, and invasion [50]. Moreover, HOTAIR establishes a positive feedback loop with STAT3, which maintains its continuous activation and upregulates resistance genes, such as ABCG2, thereby contributing to cisplatin resistance [71]. As a competing endogenous RNA (ceRNA), HOTAIR binds to miR-149-5p, lifting its suppression of HNRNPA1, which facilitates NSCLC cell invasion and EMT. Furthermore, HOTAIR’s inhibition of HOTAIRM1 through the miR-498/ABCE1 axis impedes glycolytic metabolism and the progression of tumors in non-small-cell lung cancer cells [72,73]. In addition, HOTAIR is involved in regulating key pathways such as Wnt/β-catenin and PI3K/Akt, both of which play a crucial role in tumor resistance and immune evasion [74].

### 3.4. H19

H19 is a long non-coding RNA (lncRNA) encoded by an imprinted gene, and it plays a significant role in both tumorigenesis and drug resistance in NSCLC [75]. Acting as a precursor to miR-675, H19 directly targets c-Cbl/Cbl-b, thereby promoting cell proliferation and invasion. Additionally, H19 regulates the methylation of Beclin1, which activates autophagy and enhances tumor resistance to both chemotherapy and EGFR-TKI [76]. H19 is transported to NSCLC cells via exosomes, where it binds to miR-200c, triggering the activation of the PI3K/Akt pathway and inducing resistance to gefitinib [77]. Furthermore, H19 binds competitively to miR-29b-3p, leading to an increased expression of progranulin, which in turn promotes EMT and contributes to tumor progression [78]. Moreover, H19 inhibits apoptosis in NSCLC cells by regulating multiple signaling pathways and epigenetic mechanisms, solidifying its essential role in tumor survival and resistance to drugs [79]. Finally, H19 binds to miR-29b-3p, counteracting its suppression of EMT-related genes, further increasing the proliferation and drug resistance of lung adenocarcinoma cells [80].

### 3.5. NEAT1

NEAT1 (Nuclear Enriched Abundant Transcript 1), a nuclear-enriched lncRNA, plays a significant role in promoting tumor progression in NSCLC through mechanisms involving m6A modification and ceRNA. The m6A modification mediated by METTL3 stabilizes NEAT1, enabling it to bind with miR-361-3p [81]. This binding reduces the inhibitory effect of miR-361-3p on HMGA1, which consequently promotes cell proliferation, EMT, and drug resistance [82]. In addition, NEAT1 regulates the expression of miR-181a-5p, miR-377-3p, and miR-101-3p, resulting in the increased expression of HMGB2, E2F3, and SOX9. This leads to the activation of key signaling pathways, including Wnt/β-catenin and Akt/mTOR [83]. Moreover, NEAT1 binds competitively to miR-377-3p, alleviating its inhibition of E2F3, thus promoting the proliferation and invasion of NSCLC cells [84,85]. Furthermore, NEAT1 drives tumor cell proliferation and migration via the miR-153-3p/Wnt/β-catenin axis and the miR-204/NUAK1 axis [86,87].

### 3.6. PVT1

PVT1 (Plasmacytoma Variant Translocation 1), a lncRNA located on the 8q24 chromosomal region, is closely linked to the MYC gene. In NSCLC, PVT1 regulates the expression of ITGB8 by interacting with miR-145-5p, which in turn activates the MEK/ERK signaling pathway, thereby promoting cell proliferation and migration [88]. Additionally, PVT1 binds to EZH2, inducing the methylation of the miR-497 promoter, which leads to a suppression of miR-497 expression, the upregulation of YAP1, and the activation of the NOTCH1 signaling pathway, contributing to EMT and metastasis [89]. Furthermore, PVT1 facilitates NSCLC cell proliferation, metastasis, and drug resistance through several mechanisms, including its cooperation with MYC, sequestration of miRNA, and epigenetic regulation [90]. Moreover, by sequestering miR-497, PVT1 promotes NSCLC cell proliferation and invasion by relieving the suppression of LATS2 [91]. Finally, PVT1 accelerates the malignant progression of NSCLC by modulating the expression of PGRN [78].

## 4. Autophagy

Autophagy is an evolutionarily conserved cellular degradation mechanism that plays a pivotal role in maintaining cellular homeostasis through the removal of damaged organelles and proteins [92]. This process involves intricate molecular mechanisms and regulatory networks, and its dysregulation is linked to a wide range of diseases, including cancer and neurodegenerative disorders. Consequently, targeting autophagy represents a promising therapeutic approach for addressing these conditions [93].

Recent research has highlighted the dual nature of autophagy in cancer. During the early stages, it suppresses tumor initiation by eliminating damaged organelles, whereas, in later stages, it facilitates metabolic support to tumor cells, thus contributing to drug resistance and metastasis. This dual function presents both challenges and opportunities for clinical treatment [94,95].

Specifically, certain autophagy-related genes, such as Beclin1, LC3, and ATG5, significantly influence the progression of NSCLC by regulating processes like cell death, metabolic reprogramming, and adaptation to the microenvironment, thereby laying the groundwork for developing novel therapeutic strategies [96]. Furthermore, autophagy is involved in regulating cellular metabolism, oxidative stress, and immune responses, where it can act as a tumor suppressor in some contexts, while promoting cell survival and resistance in the tumor microenvironment [97].

To ensure the reliability and reproducibility of results, autophagy research must follow standardized experimental protocols and guidelines for data interpretation, providing authoritative references for both basic and clinical studies [98]. Finally, the intricate nature of the autophagy regulatory network positions it as a promising target for cancer treatment, with the combined use of autophagy inhibitors and chemotherapy potentially enhancing the therapeutic effectiveness in NSCLC [99].

### 4.1. Types of Autophagy

Autophagy consists of three primary types, namely, macroautophagy, microautophagy, and chaperone-mediated autophagy, each of which plays distinct roles in the context of NSCLC. Among these, macroautophagy is the most prevalent form and involves the formation of double-membrane autophagosomes, followed by fusion with lysosomes [100]. This pathway acts as the central mechanism for cellular stress response in NSCLC, and disruptions in its regulation are strongly associated with tumor progression [100,101]. Key molecules such as ULK1, Beclin1, and LC3 are critical for macroautophagy, whereas microautophagy facilitates the direct degradation of cellular material through the invagination of the lysosomal membrane [100]. In contrast, CMA recognizes specific proteins through the involvement of Hsc70 and LAMP-2A, each playing a unique role in maintaining cellular homeostasis [102]. CMA targets proteins with KFERQ-like sequences for selective degradation, thereby influencing stress responses, metabolic control, and NSCLC cell survival [103]. Targeting CMA through modulation could offer new therapeutic strategies for treatment [104]. These three autophagy pathways work synergistically to uphold cellular homeostasis in response to stress and disease through mutual interactions and compensatory mechanisms. Further investigation is essential to fully understand how these types of autophagy coordinate within the NSCLC microenvironment [105]. Moreover, these autophagy mechanisms are crucial for processes such as embryonic development, cellular differentiation, and tissue remodeling, with macroautophagy playing a particularly dominant role in NSCLC [106]. Understanding the molecular mechanisms and regulatory networks that underlie these different autophagy types provides a strong theoretical foundation for the development of autophagy-based targeted therapies for NSCLC [107].

### 4.2. Mechanisms of Autophagy

**Figure 3:**
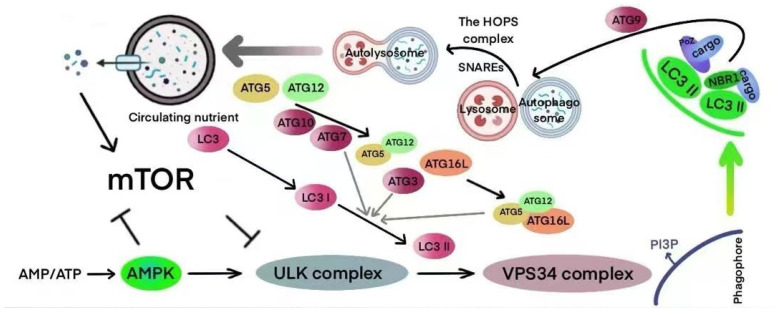
Diagram illustrating the mechanisms of autophagy.

The molecular mechanisms underlying autophagy involve five key stages, namely, initiation, elongation, closure, fusion, and degradation, all of which depend on the coordinated function of several core proteins and signaling pathways (Figure 3). Autophagy is triggered by fluctuations in cellular energy status, with the mTOR–AMPK–ULK1 axis serving as the central regulatory hub. The ULK1 complex plays a crucial role in initiating the process, becoming activated through AMPK signaling under energy stress and inhibited by mTOR when nutrients are abundant. Once initiated, autophagy proceeds through the lipidation and membrane integration of LC3, leading to the formation and maturation of autophagosomes. These vesicles then fuse with lysosomes in a highly coordinated manner to form autolysosomes, where cellular components are degraded and recycled. This tightly regulated cascade enables cells to maintain homeostasis and adapt to changing metabolic and environmental conditions. Upon activation, the complex recruits the Beclin1-VPS34 complex, which begins the formation of autophagosomes, a critical process for the stress response in NSCLC cells [108]. The formation of autophagosomes requires the cooperative action of proteins such as ATG5-ATG12 and LC3, enabling their extension, maturation, and fusion with lysosomes, which in turn regulates the metabolism and survival of NSCLC cells [109]. The complex regulatory network of autophagy plays a multifaceted role in the tumor microenvironment, EMT, and ROS regulation, offering new avenues for targeted therapies in NSCLC [110]. Additionally, autophagy is regulated by metabolic signals, including AMPK and mTOR, which influence tumor behavior in NSCLC through various mechanisms:

EMT and metastasis: Autophagy regulates proteins related to EMT, such as E-cadherin, which enhances the ability of NSCLC cells to invade and metastasize [111]. lncRNA BCAR4 promotes the proliferation, invasion, and metastasis of NSCLC cells by facilitating epithelial–mesenchymal transition. BCAR4 upregulation was associated with decreased E-cadherin and increased mesenchymal markers, indicating its pro-EMT function [112]. Similarly, TGF-β-induced lncRNA TBUR1 enhances EMT and metastasis in lung adenocarcinoma by stabilizing GRB2 mRNA via hnRNPC-mediated binding, thereby activating downstream oncogenic signaling [113].

Tumor microenvironment: By modulating immune cell activity, stromal metabolism, and the secretion of cytokines, autophagy plays a crucial role in shaping the NSCLC microenvironment and promoting immune evasion [114].

ROS regulation: Through the elimination of reactive oxygen species (ROS), autophagy helps mitigate oxidative stress, thereby safeguarding NSCLC cells from apoptosis or triggering cell death via ROS accumulation [115].

## 5. The Dual Regulatory Effect of Autophagy in Non-Small-Cell Lung Cancer

Autophagy plays a variable role in NSCLC, with its function influenced by the disease stage and the surrounding microenvironment. Depending on these factors, autophagy can exhibit both tumor-suppressive and tumor-promoting effects, highlighting its complex involvement in cancer progression [116].

### 5.1. The Tumor-Inhibiting Role of Autophagy in Non-Small-Cell Lung Cancer

Autophagy has been shown to suppress genomic instability and prevent tumor formation in the early stages of NSCLC by removing damaged organelles and abnormal proteins. The activation of autophagy-related proteins, such as Beclin1 and LC3, facilitates cell apoptosis, establishing a molecular foundation for its tumor-suppressive effects [117]. In a similar study, autophagy plays a tumor-suppressive role in early-stage NSCLC by regulating oxidative stress and DNA repair mechanisms [118]. Moreover, the downregulation of genes associated with this process may accelerate tumor progression, highlighting the potential therapeutic benefits of activating autophagy [119].

### 5.2. The Tumor-Promoting Effects of Autophagy in Non-Small-Cell Lung Cancer

In advanced NSCLC, autophagy enables tumor cells to withstand stressful conditions, including hypoxia and nutrient deprivation, by supplying essential metabolic substrates and energy [120]. This mechanism contributes to resistance against both chemotherapy and targeted therapies. Furthermore, combining autophagy inhibitors with chemotherapy and targeted drugs has been shown to significantly improve treatment outcomes [55]. The activation of autophagy-related proteins, such as ATG7 and Beclin1, assists NSCLC cells in adapting to harsh microenvironments [121]. Elevated levels of autophagy are strongly correlated with tumor progression and poor prognosis [122]. In further investigations, autophagy-related lncRNAs, such as ABALON, play an important role in regulating autophagy, significantly influencing the survival of NSCLC patients. A heightened autophagy state is closely associated with chemotherapy resistance, underscoring the therapeutic potential of targeting autophagy [123]. Liu’s research team found that lncRNA XIST promotes autophagy through the regulation of ATG7, enhancing resistance to cisplatin in NSCLC cells. Knocking down XIST inhibits autophagy and increases chemotherapy sensitivity, offering a promising new target for overcoming drug resistance [124].

## 6. lncRNAs and Autophagy in Non-Small-Cell Lung Cancer

LncRNAs and autophagy have gained significant attention in recent years due to their intricate roles in regulating NSCLC. These molecules affect tumor cell proliferation, apoptosis, invasion, and drug resistance by interacting through complex molecular networks, providing key theoretical insights for advancing precision medicine [125]. An analysis of the TCGA database identified seven autophagy-related lncRNAs, including ABALON, NKILA, and AC092171.2, which were used to construct a prognostic model for NSCLC patients [126]. ABALON promotes autophagosome formation and chemotherapy resistance by upregulating ATG7 expression. The model predicted a 3-year survival rate with an AUC value of 0.625 and a sensitivity of 78.2%, presenting a reliable tool for developing personalized treatment strategies [127]. Zhang’s team further identified that autophagy-related lncRNAs (ARlncRNAs) regulate various stages of autophagic flow, including initiation, elongation, and fusion, through a competitive endogenous RNA (ceRNA) mechanism or direct interaction with autophagy-related proteins. This regulation plays a significant role in the survival and drug resistance of NSCLC cells, positioning these lncRNAs as promising candidates for diagnostic biomarkers and therapeutic targets [128]. A detailed summary revealed that lncRNAs such as LUCAT1 and SNHG7 fine-tune autophagy in NSCLC through mechanisms including miRNA competition (e.g., miR-514a-3p), protein interactions (e.g., binding to BECN1), and epigenetic regulation [129]. This regulation profoundly influences tumor progression and chemotherapy response, underscoring the potential for clinical application by targeting these lncRNAs [130]. Collectively, these studies suggest that autophagy-related lncRNAs are strongly linked to the onset and progression of non-small-cell lung cancer, making them valuable diagnostic biomarkers and therapeutic targets [131].

It was further highlighted that lncRNAs such as HOTAIR, MALAT1 and XIST play a crucial role in regulating the transcription and translation of key autophagy-related genes, including ATG7, LC3B and ULK1. These lncRNAs influence various processes such as metabolic reprogramming, oxidative stress responses, and microenvironmental adaptation in NSCLC cells [132]. Their functional interaction is vital for advancing our understanding of the molecular pathological mechanisms underlying NSCLC [73]. Li’s team found that the overexpression of lncRNA XIST promotes the formation of autophagosomes via the miR-186-5p/ATG7 axis, which contributes to enhanced resistance to cisplatin in NSCLC cells. Experimental validation confirmed that silencing XIST reduces the LC3-II/LC3-I ratio, inhibits autophagic flux, and increases sensitivity to chemotherapy (with a reduction in IC50 by approximately 40%), highlighting XIST as a promising target for overcoming drug resistance [133]. Furthermore, lncRNA BLACAT1 activates autophagy through the miR-17/ATG7 pathway, which significantly improves the survival rate of NSCLC cells treated with cisplatin [134]. Suppressing BLACAT1 downregulates ATG7 expression, decreases autophagosome numbers, and enhances chemotherapy sensitivity by nearly twofold, emphasizing its potential as a therapeutic target to reverse drug resistance [135].

The studies outlined above highlight the dual role of lncRNAs in the initiation, progression, and therapeutic response of NSCLC, as they can either activate or inhibit autophagy [136]. A comprehensive analysis of their regulatory mechanisms not only aids in clarifying the molecular pathological processes of NSCLC but also forms the theoretical basis for developing precision-targeted therapies aimed at lncRNAs. However, the relationship between lncRNAs and autophagy is influenced by factors such as tumor stage, microenvironment, and treatment context [137]. Therefore, further research is essential to delineate their specific functions at different stages of the disease [138].

### 6.1. The Suppressive Role of Autophagy-Related lncRNAs in Autophagy in Non-Small-Cell Lung Cancer

Certain lncRNAs modulate autophagy levels in NSCLC cells by either downregulating the expression of autophagy-related genes or inhibiting the formation of autophagosomes. This regulation, in turn, influences tumor proliferation, invasion, and the response to therapy [125]. For instance, lncRNA NBAT1 binds to the promoter region of ATG7, suppressing its transcriptional activity and significantly lowering autophagy levels in NSCLC cells (resulting in about a 50% reduction in LC3-II accumulation) [139]. This reduction inhibits both tumor cell proliferation and migration. Furthermore, a low expression of NBAT1 is associated with poorer prognosis in NSCLC patients, suggesting that NBAT1 could act as a potential tumor suppressor [113]. Sun’s team found that silencing lncRNA XIST leads to the downregulation of ATG7 and Beclin1, inhibits autophagosome formation, reduces autophagic activity in NSCLC cells, and enhances the cytotoxic effects of cisplatin (with an approximately 35% increase in apoptosis) [140]. These findings indicate that the mechanism by which XIST suppresses autophagy plays an important role in reversing chemotherapy resistance [140]. Additionally, lncRNA NKILA competitively binds to miR-21, indirectly downregulating the expression of LC3B and ATG5, thus inhibiting autophagic flux in NSCLC cells [141]. A high expression of NKILA is linked to longer overall survival and holds significant prognostic value in predictive models (HR = 0.62, *p* < 0.05) [142].

#### 6.1.1. Autophagy-Related lncRNAs Inhibit Autophagy and the Pathogenesis of Non-Small-Cell Lung Cancer

Certain lncRNAs can inhibit autophagy, resulting in tumor-suppressive effects that help restrict the initiation and progression of NSCLC [131]. For instance, under specific conditions, lncRNA HOTAIR downregulates autophagy-related proteins via the miR-613/ATG5 axis, causing a significant reduction in autophagosome formation in NSCLC cells (with approximately a 60% decrease in autophagic flux) [143]. This reduction, in turn, suppresses tumor cell proliferation and migration [144]. The low expression of HOTAIR is linked to a better prognosis, suggesting its potential role as a tumor suppressor in the early stages of NSCLC [145]. Moreover, lncRNA ACTA2-AS recruits EZH2 to the TSC2 gene promoter, suppressing TSC2 expression, inhibiting autophagy, promoting apoptosis in cisplatin-resistant NSCLC cells, and limiting tumor progression. Additionally, experiments have shown that the overexpression of ACTA2-AS1 can restore autophagy and promote apoptosis, presenting a potential target for early therapeutic intervention [146].

These studies suggest that lncRNAs may play a role in reducing tumor malignancy during the early stages of NSCLC by inhibiting autophagic flux [136]. This occurs through mechanisms such as the clearance of damaged organelles, the stabilization of genomic integrity, and the induction of apoptosis [147]. However, the tumor-suppressive effects of these lncRNAs may be attenuated by changes in the tumor microenvironment, including conditions like hypoxia or nutrient deprivation. This underscores the necessity for further research to better understand their stage-specific roles and mechanisms [148].

#### 6.1.2. Autophagy-Related lncRNAs Inhibit Autophagy and Promote the Pathogenesis of Non-Small-Cell Lung Cancer

Certain lncRNAs may inhibit autophagy, potentially leading to a more aggressive phenotype in NSCLC. For example, specific ARlncRNAs, such as LINC00460, interact with the ATG12 promoter region, thereby suppressing its transcriptional activity and preventing the formation of autophagosomes [149]. This process enables NSCLC cells to escape autophagy-induced apoptosis, subsequently facilitating tumor proliferation and invasion [114]. Experimental findings indicated that silencing LINC00460 restores autophagy and decreases cell viability by approximately 30% [150]. These lncRNAs, by disrupting the maturation of autophagosomes or their fusion with lysosomes, enhance the survival of NSCLC cells in response to chemotherapy or oxidative stress, particularly in advanced tumor stages [151]. These mechanisms highlight the tumor-promoting role of lncRNAs inhibiting autophagy, which is closely associated with the tumor microenvironment and therapeutic context, underlining the importance of further investigation into their molecular mechanisms in relation to clinical staging [152,153].

### 6.2. The Promoting Effect of Autophagy-Related lncRNAs on Autophagy in Non-Small-Cell Lung Cancer

Certain lncRNAs contribute to increased autophagy levels in NSCLC cells by either upregulating the expression of genes related to autophagy or promoting the formation of autophagosomes. These processes, in turn, influence tumor survival, resistance to treatment, and metastasis [154]. LncRNA BLACAT1 boosts autophagic flux through the miR-17/ATG7 pathway, thereby enhancing NSCLC cell survival during cisplatin treatment, with a 45% increase in survival rate. Moreover, the experimental inhibition of BLACAT1 was shown to reduce the levels of ATG7 and LC3-II, decrease autophagosome numbers, and markedly improve chemotherapy sensitivity, as evidenced by a 50% decrease in IC50 values [134]. In a similar vein, lncRNA LUCAT1 activates autophagy via the miR-514a-3p/ULK1 axis, which significantly enhances autophagic flux in NSCLC cells, causing a twofold increase in the LC3-II/LC3-I ratio [155]. This, in turn, leads to chemotherapy resistance. Additionally, SNHG7 contributes to cisplatin resistance by upregulating the expression of LC3B and BECN1 [156]. Interestingly, silencing LUCAT1 or SNHG7 was found to reverse the resistance phenotype, suggesting potential targets for therapeutic intervention [157]. Furthermore, high expression levels of lncRNA SIX1 promote the formation of autophagosomes through the miR-186-5p/ATG7 axis, thus enhancing NSCLC cell resistance to cisplatin. Silencing SIX1 significantly reduces autophagic activity (with a 60% decrease in autophagosome numbers) and improves chemotherapy sensitivity, providing a molecular foundation for the reversal of drug resistance [158].

#### 6.2.1. Autophagy-Related lncRNAs Activate Autophagy and Suppress the Pathogenesis of Non-Small-Cell Lung Cancer

Under certain conditions, lncRNAs may play a role in inhibiting tumor progression by activating autophagy. Some ARlncRNAs, such as LINC00641, increase the expression of Beclin1 and ATG5 via the ceRNA mechanism, which in turn enhances autophagic flux in NSCLC cells, with a 70% rise in LC3-II accumulation [155]. This process induces apoptosis and impedes the early stages of tumorigenesis. Further experimental findings showed that the overexpression of LINC00641 significantly lowered the tumor formation rate by approximately 40% [159]. These lncRNAs promote the formation of autophagosomes and their fusion with lysosomes, facilitating the clearance of damaged organelles and abnormal proteins, thereby preserving genomic stability and limiting the malignant transformation of NSCLC cells. However, the anti-tumor effects of these lncRNAs may be diminished in advanced tumors or under stressful microenvironments [160]. Therefore, further studies are necessary to investigate their stage-specific functions and regulatory mechanisms.

#### 6.2.2. Autophagy-Related lncRNAs Activate Autophagy and Promote the Pathogenesis of Non-Small-Cell Lung Cancer

Additional research suggests that lncRNAs promote the malignant progression and drug resistance of NSCLC through the activation of autophagy. LncRNA SNHG7 increases autophagic activity in NSCLC cells by upregulating the expression of LC3B and BECN1, resulting in an approximately 80% increase in autophagic flux, which subsequently contributes to cisplatin resistance [156]. When SNHG7 was silenced, autophagy levels decreased, and chemotherapy sensitivity increased by about 2.5 times, highlighting the critical role of SNHG7 as a tumor-promoting factor [156]. Likewise, the high expression of lncRNA HOTAIR activates autophagy via the miR-6807-5p/Egr1 axis, leading to enhanced NSCLC cell proliferation, invasion, and resistance to chemotherapy. Experimental data revealed that the overexpression of HOTAIR resulted in a twofold increase in the number of autophagosomes and a 50% improvement in cell survival, underscoring its pro-tumor function in the later stages of NSCLC [161].

These lncRNAs facilitate the formation, maturation, and fusion of autophagosomes with lysosomes, thereby supplying energy and metabolic substrates to NSCLC cells under stress conditions, such as nutrient deprivation, oxidative stress, or chemotherapy-induced stress [162]. As a result, tumor survival, metastasis, and drug resistance are enhanced. The studies mentioned above suggest that targeting autophagy-promoting lncRNAs, through approaches like RNA interference or CRISPR technology, can significantly reverse the malignant phenotype of NSCLC, offering important directions for the development of new therapeutic strategies [163].

## 7. Clinical Significance of lncRNAs in Autophagy and Resistance in Non-Small-Cell Lung Cancer

lncRNAs play a crucial role in regulating autophagy and demonstrate considerable clinical potential in diagnosing, prognosticating, and treating resistance in NSCLC. This offers new possibilities for the advancement of precision medicine [87]. A prognostic model using 14 autophagy-related lncRNAs (such as ABALON, NKILA, AC092171.2, and others) was capable of accurately predicting the 3-year survival rate of NSCLC patients (AUC = 0.625; sensitivity 78.2%; specificity 72.5%) [126]. Through the regulation of autophagy-related genes (including ATG7 and LC3B), the model affects tumor progression and chemotherapy resistance, providing a robust tool for clinical risk stratification and the design of personalized treatment plans [126]. ARlncRNAs function as both biomarkers and drug targets. They regulate autophagic flux through the ceRNA mechanism or by interacting directly with autophagy proteins, significantly influencing the therapeutic outcomes and prognosis of NSCLC. Targeting these lncRNAs (e.g., via siRNA or ASO technology) offers significant potential for improving patient survival [164].

LncRNA BLACAT1 enhances autophagy through the miR-17/ATG7 pathway, leading to the increased resistance of NSCLC cells to cisplatin (with IC50 approximately doubling). The suppression of BLACAT1 significantly lowers autophagy levels, reverses the resistance phenotype, and enhances chemotherapy sensitivity by about 50%, providing potential new targets for overcoming drug resistance [165]. The role of lncRNAs, including LUCAT1 and SNHG7, in inhibiting chemotherapy and targeted therapies in NSCLC through the activation of autophagy [151]. Intervening with these lncRNAs using strategies such as RNA interference, small molecule inhibitors, or nanoparticle delivery systems can significantly improve therapeutic outcomes. Preclinical studies have shown that silencing LUCAT1 results in a reduction in tumor burden by approximately 40% [52]. Silencing lncRNA XIST enhances cisplatin sensitivity in NSCLC cells by inhibiting ATG7 and autophagic flux, with the apoptosis rate increasing by about 35%. The potential of XIST as a target for reversing drug resistance has been confirmed through various in vitro and in vivo experiments [166]. LncRNA HOTAIR plays a crucial role in determining the prognosis of NSCLC by regulating autophagy and drug resistance-related pathways, such as the miR-613/ATG7 axis [167]. High expression levels of HOTAIR are linked to chemotherapy failure and a shortened survival time (HR = 1.85; *p* < 0.01), highlighting its dual role as both a prognostic marker and a potential therapeutic target. Clinical studies indicate that patients exhibiting high HOTAIR expression experience a reduction of approximately 30% in the objective response rate (ORR) [168]. In summary, lncRNAs regulate autophagy and hold substantial clinical value in diagnosing, prognosticating, and treating drug resistance in NSCLC [169]. Future research should prioritize the development of intervention strategies targeting lncRNAs, such as CRISPR/Cas9-based gene editing or nanoparticle drug delivery systems, while conducting multi-center clinical trials to evaluate their efficacy and safety, ultimately enhancing treatment outcomes and prolonging patient survival in NSCLC [170].

## 8. Conclusions and Perspectives

The regulatory role of lncRNAs in autophagy and drug resistance in NSCLC has revealed their complex function as essential molecular switches. By interacting with autophagy-related genes, miRNAs, and proteins, lncRNAs play dual roles in the initiation, progression, and therapeutic response of NSCLC. While some lncRNAs inhibit autophagy to suppress early tumor growth or increase chemotherapy sensitivity, others activate autophagy, thereby promoting tumor cell survival and resistance under stress conditions. These mechanisms involve ceRNA networks, epigenetic modifications, and signaling pathway regulation, providing fresh insights into the molecular pathology of NSCLC. Additionally, the potential of lncRNAs as diagnostic markers, prognostic indicators, and therapeutic targets has been substantiated in multiple studies, highlighting their promising applications in the development of prognostic models, the reversal of drug resistance, and the direction of personalized treatments.

The study of the interaction between lncRNAs and autophagy continues to face several challenges. The role of lncRNAs is influenced by factors such as tumor stage, the microenvironment, and treatment context, emphasizing the need for more systematic investigations to uncover their stage-specific mechanisms. The complexity of the lncRNA regulatory network requires the integration of multi-omics data (e.g., transcriptomics and epigenomics) to achieve a comprehensive understanding of its regulatory landscape. Intervention strategies targeting lncRNAs, including RNA interference, CRISPR/Cas9 gene editing, and nanoparticle delivery systems, involve technical hurdles related to specificity, delivery efficiency, and safety in clinical translation. In the future, multi-center clinical trials will be crucial for validating the diagnostic and therapeutic potential of lncRNAs, especially when combined with artificial intelligence and precision medicine approaches. These advancements offer promising potential to enhance the survival and quality of life of NSCLC patients, thereby providing a strong foundation for the development of novel diagnostic and therapeutic strategies.

## Data Availability

All data presented in this study are included within the paper.

## Abbreviations

The following abbreviations are used in this manuscript:

Abbreviation	Full Term
NSCLC	Non-Small-Cell Lung Cancer
lncRNA	Long Non-Coding RNA
ceRNA	Competing Endogenous RNA
EMT	Epithelial–Mesenchymal Transition
FDA	Food and Drug Administration
EGFR-TKI	Epidermal Growth Factor Receptor-Tyrosine Kinase Inhibitor
STAT3	Signal Transducer and Activator of Transcription 3
mTOR	Mechanistic Target of Rapamycin
AMPK	AMP-Activated Protein Kinase
ULK1	Unc-51 Like Autophagy Activating Kinase 1
LC3	Microtubule-Associated Protein 1 Light Chain 3
ATG	Autophagy-Related Gene
CMA	Chaperone-Mediated Autophagy
ROS	Reactive Oxygen Species
PSA	Prostate-Specific Antigen
HOTAIR	HOX Transcript Antisense RNA
MALAT1	Metastasis-Associated Lung Adenocarcinoma Transcript 1
PVT1	Plasmacytoma Variant Translocation 1
XIST	X-Inactive Specific Transcript
BLACAT1	Bladder Cancer-Associated Transcript 1
LUCAT1	Lung Cancer-Associated Transcript 1
SNHG7	Small Nucleolar RNA Host Gene 7
TCGA	The Cancer Genome Atlas
ORR	Objective Response Rate
IC50	Half Maximal Inhibitory Concentration

## References

[1] Suster D.I., Mino-Kenudson M. (2020). Molecular Pathology of Primary Non-small Cell Lung Cancer. Arch. Med. Res..

[2] Pelosi G., Barbareschi M., Cavazza A., Graziano P., Rossi G., Papotti M. (2015). Large cell carcinoma of the lung: A tumor in search of an author. A clinically oriented critical reappraisal. Lung Cancer.

[3] Garg P., Singhal S., Kulkarni P., Horne D., Malhotra J., Salgia R., Singhal S.S. (2024). Advances in Non-Small Cell Lung Cancer: Current Insights and Future Directions. J. Clin. Med..

[4] Bray F., Laversanne M., Sung H., Ferlay J., Siegel R.L., Soerjomataram I., Jemal A. (2024). Global cancer statistics 2022: Globocan estimates of incidence and mortality worldwide for 36 cancers in 185 countries. CA Cancer J. Clin..

[5] Leiter A., Veluswamy R.R., Wisnivesky J.P. (2023). The global burden of lung cancer: Current status and future trends. Nat. Rev. Clin. Oncol..

[6] Sosa Iglesias V., Giuranno L., Dubois L.J., Theys J., Vooijs M. (2018). Drug Resistance in Non-Small Cell Lung Cancer: A Potential for NOTCH Targeting?. Front. Oncol..

[7] Min H.Y., Lee H.Y. (2021). Mechanisms of resistance to chemotherapy in non-small cell lung cancer. Arch. Pharmacal Res..

[8] Chen Y., Wu S., Han Y., Shi H., Yuan J., Cui W. (2024). LncRNA SH3PXD2A-AS1 facilitates cisplatin resistance in non-small cell lung cancer by regulating FOXM1 succinylation. BMC Cancer.

[9] Vitto V.A.M., Bianchin S., Zolondick A.A., Pellielo G., Rimessi A., Chianese D., Yang H., Carbone M., Pinton P., Giorgi C. (2022). Molecular Mechanisms of Autophagy in Cancer Development, Progression, and Therapy. Biomedicines.

[10] Debnath J., Gammoh N., Ryan K.M. (2023). Autophagy and autophagy-related pathways in cancer. Nat. Rev. Mol. Cell Biol..

[11] Li X., He S., Ma B. (2020). Autophagy and autophagy-related proteins in cancer. Mol. Cancer.

[12] Han J., Goldstein L.A., Hou W., Chatterjee S., Burns T.F., Rabinowich H. (2018). HSP90 inhibition targets autophagy and induces a CASP9-dependent resistance mechanism in NSCLC. Autophagy.

[13] Guo W., Du K., Luo S., Hu D. (2022). Recent Advances of Autophagy in Non-Small Cell Lung Cancer: From Basic Mechanisms to Clinical Application. Front. Oncol..

[14] Yu M., Li H., Wu Y., Liu P., Xu Q., Zhang Y. (2025). Autophagy-associated ncRNAs in lung cancer: From drug resistance to therapeutic targets. Int. J. Biol. Macromol..

[15] Ratti M., Lampis A., Ghidini M., Salati M., Mirchev M.B., Valeri N., Hahne J.C. (2020). MicroRNAs (miRNAs) and Long Non-Coding RNAs (lncRNAs) as New Tools for Cancer Therapy: First Steps from Bench to Bedside. Target. Oncol..

[16] Statello L., Guo C.J., Chen L.L., Huarte M. (2021). Gene regulation by long non-coding RNAs and its biological functions. Nat. Rev. Mol. Cell Biol..

[17] Lin W., Zhou Q., Wang C.Q., Zhu L., Bi C., Zhang S., Wang X., Jin H. (2020). LncRNAs regulate metabolism in cancer. Int. J. Biol. Sci..

[18] Fu J., Yu L., Yan H., Tang S., Wang Z., Dai T., Chen H., Zhang S., Hu H., Liu T. (2023). LncRNAs in non-small cell lung cancer: Novel diagnostic and prognostic biomarkers. Front. Mol. Biosci..

[19] Al-Tobasei R., Paneru B., Salem M. (2016). Genome-Wide Discovery of Long Non-Coding RNAs in Rainbow Trout. PLoS ONE.

[20] Yi Q., Feng J., Lan W., Shi H., Sun W., Sun W. (2024). CircRNA and lncRNA-encoded peptide in diseases, an update review. Mol. Cancer.

[21] Djebali S., Davis C.A., Merkel A., Dobin A., Lassmann T., Mortazavi A., Tanzer A., Lagarde J., Lin W., Schlesinger F. (2012). Landscape of transcription in human cells. Nature.

[22] Bhat S.A., Ahmad S.M., Mumtaz P.T., Malik A.A., Dar M.A., Urwat U., Shah R.A., Ganai N.A. (2016). Long non-coding RNAs: Mechanism of action and functional utility. Non-Coding RNA Res..

[23] Kopp F., Mendell J.T. (2018). Functional Classification and Experimental Dissection of Long Noncoding RNAs. Cell.

[24] St Laurent G., Wahlestedt C., Kapranov P. (2015). The Landscape of long noncoding RNA classification. Trends Genet..

[25] Lin Y.H. (2020). Crosstalk of lncRNA and Cellular Metabolism and Their Regulatory Mechanism in Cancer. Int. J. Mol. Sci..

[26] Derrien T., Johnson R., Bussotti G., Tanzer A., Djebali S., Tilgner H., Guernec G., Martin D., Merkel A., Knowles D.G. (2012). The GENCODE v7 catalog of human long noncoding RNAs: Analysis of their gene structure, evolution, and expression. Genome Res..

[27] Herman A.B., Tsitsipatis D., Gorospe M. (2022). Integrated lncRNA function upon genomic and epigenomic regulation. Mol. Cell.

[28] Hombach S., Kretz M. (2016). Non-coding RNAs: Classification, Biology and Functioning. Adv. Exp. Med. Biol..

[29] Mattick J.S., Amaral P.P., Carninci P., Carpenter S., Chang H.Y., Chen L.L., Chen R., Dean C., Dinger M.E., Fitzgerald K.A. (2023). Long non-coding RNAs: Definitions, functions, challenges and recommendations. Nat. Rev. Mol. Cell Biol..

[30] Lennox K.A., Behlke M.A. (2016). Cellular localization of long non-coding RNAs affects silencing by RNAi more than by antisense oligonucleotides. Nucleic Acids Res..

[31] Carlevaro-Fita J., Polidori T., Das M., Navarro C., Zoller T.I., Johnson R. (2019). Ancient exapted transposable elements promote nuclear enrichment of human long noncoding RNAs. Genome Res..

[32] Quinodoz S., Guttman M. (2014). Long noncoding RNAs: An emerging link between gene regulation and nuclear organization. Trends Cell Biol..

[33] Naganuma T., Hirose T. (2013). Paraspeckle formation during the biogenesis of long non-coding RNAs. RNA Biol..

[34] Song Z., Lin J., Li Z., Huang C. (2021). The nuclear functions of long noncoding RNAs come into focus. Non-Coding RNA Res..

[35] Hussein M.A., Valinezhad K., Adel E., Munirathinam G. (2024). MALAT-1 Is a Key Regulator of Epithelial-Mesenchymal Transition in Cancer: A Potential Therapeutic Target for Metastasis. Cancers.

[36] Naseer Q.A., Malik A., Zhang F., Chen S. (2024). Exploring the enigma: History, present, and future of long non-coding RNAs in cancer. Discov. Oncol..

[37] Tay Y., Rinn J., Pandolfi P.P. (2014). The multilayered complexity of ceRNA crosstalk and competition. Nature.

[38] Al-Imam M.J., Hussein U.A., Sead F.F., Faqri A.M.A., Mekkey S.M., Khazel A.J., Almashhadani H.A. (2023). The interactions between DNA methylation machinery and long non-coding RNAs in tumor progression and drug resistance. DNA Repair.

[39] Wang C.J., Shi S.B., Tian J., Xu J., Niu Z.X. (2017). lncRNA MALAT1, HOTTIP and PVT1 as predictors for predicting the efficacy of GEM based chemotherapy in first-line treatment of pancreatic cancer patients. Oncotarget.

[40] Badowski C., He B., Garmire L.X. (2022). Blood-derived lncRNAs as biomarkers for cancer diagnosis: The Good, the Bad and the Beauty. NPJ Precis. Oncol..

[41] Nagarajah S., Saleh Q.W., Rasmussen M., Tepel M. (2024). Long non-coding RNA MGAT3 in kidney transplant recipients with immunoglobulin A nephropathy. J. Nephrol..

[42] Galbiati S., Bettiga A., Colciago G., Senti C., Trevisani F., Villa G., Marzinotto I., Ghidini M., Passalacqua R., Montorsi F. (2024). The long noncoding RNA SUMO1P3 as urinary biomarker for monitoring bladder cancer progression. Front. Oncol..

[43] Beylerli O., Gareev I., Sufianov A., Ilyasova T., Guang Y. (2022). Long noncoding RNAs as promising biomarkers in cancer. Non-Coding RNA Res..

[44] Qiao Y., Tian X., Li S., Niu H. (2024). Identification and experimental validation of a sialylation-related long noncoding RNA signature for prognosis of bladder cancer. BMC Urol..

[45] Zhang Y. (2024). LncRNA-encoded peptides in cancer. J. Hematol. Oncol..

[46] Nemeth K., Bayraktar R., Ferracin M., Calin G.A. (2024). Non-coding RNAs in disease: From mechanisms to therapeutics. Nat. Rev. Genet..

[47] Rodríguez-Malavé N.I., Rao D.S. (2016). Long noncoding RNAs in hematopoietic malignancies. Brief. Funct. Genom..

[48] Tao Y., Liu J., Qiu W., Li Y. (2025). LncRNA MANCR is downregulated in non-small cell lung cancer and predicts poor survival. Discov. Oncol..

[49] Miao X., Xi W., Bao Y. (2023). LncRNA RP11-58O9.2 predicts poor prognosis and promotes progression of non-small cell lung cancer. J. Int. Med. Res..

[50] Liang H., Peng J. (2022). LncRNA HOTAIR promotes proliferation, invasion and migration in NSCLC cells via the CCL22 signaling pathway. PLoS ONE.

[51] Chi X., Feng L., Wang L., Yu S., Wei M., Zhang Q., Liu X., Shao S. (2025). Downregulation of lncRNA MNX1-AS1 promotes the ferroptosis and apoptosis of non-small cell lung cancer. Int. J. Med. Sci..

[52] Fang F., Zhao M., Meng J., He J., Yang C., Wang C., Wang J., Xie S., Jin X., Shi W. (2025). Upregulation of TTYH3 by lncRNA LUCAT1 through interacting with ALYREF facilitates the metastasis in non-small cell lung cancer. Cancer Biol. Ther..

[53] Pan H., Yu T., Sun L., Chai W., Liu X., Yan M. (2020). LncRNA FENDRR-mediated tumor suppression and tumor-immune microenvironment changes in non-small cell lung cancer. Transl. Cancer Res..

[54] Xiong Z., Han Z., Pan W., Zhu X., Liu C. (2023). Correlation between chromatin epigenetic-related lncRNA signature (CELncSig) and prognosis, immune microenvironment, and immunotherapy in non-small cell lung cancer. PLoS ONE.

[55] Wang M., Fu Y., Zhong C., Gacche R.N., Wu P.J.H. (2023). Long non-coding RNA and Evolving drug resistance in lung cancer. Heliyon.

[56] Xia R., Geng G., Yu X., Xu Z., Guo J., Liu H., Li N., Li Z., Li Y., Dai X. (2021). LINC01140 promotes the progression and tumor immune escape in lung cancer by sponging multiple microRNAs. J. Immunother. Cancer.

[57] Chatterjee M., Nag S., Gupta S., Mukherjee T., Shankar P., Parashar D., Maitra A., Das K. (2025). MicroRNAs in lung cancer: Their role in tumor progression, biomarkers, diagnostic, prognostic, and therapeutic relevance. Discov. Oncol..

[58] Wang Y., Li M., Zhang L., Chen Y., Ha M. (2022). LINC01140 inhibits nonsmall cell lung cancer progression and cisplatin resistance through the miR-4742-5p/TACC1 axis. J. Biochem. Mol. Toxicol..

[59] He L., Zhao X., He L. (2020). LINC01140 Alleviates the Oxidized Low-Density Lipoprotein-Induced Inflammatory Response in Macrophages via Suppressing miR-23b. Inflammation.

[60] Li X. (2022). LINC01140 Targeting miR-452-5p/RGS2 Pathway to Attenuate Breast Cancer Tumorigenesis. Dis. Markers.

[61] Gencel-Augusto J., Wu W., Bivona T.G. (2023). Long Non-Coding RNAs as Emerging Targets in Lung Cancer. Cancers.

[62] Li S., Ma F., Jiang K., Shan H., Shi M., Chen B. (2018). Long non-coding RNA metastasis-associated lung adenocarcinoma transcript 1 promotes lung adenocarcinoma by directly interacting with specificity protein 1. Cancer Sci..

[63] Tetik Vardarlı A., Ozgur S., Goksel T., Korba K., Karakus H.S., Asık A., Pelit L., Gunduz C. (2023). Conversion of specific lncRNAs to biomarkers in exhaled breath condensate samples of patients with advanced stage non-small-cell lung cancer. Front. Genet..

[64] Zhao Y., Zhou L., Li H., Sun T., Wen X., Li X., Meng Y., Li Y., Liu M., Liu S. (2021). Nuclear-Encoded lncRNA MALAT1 Epigenetically Controls Metabolic Reprogramming in HCC Cells through the Mitophagy Pathway. Mol. Ther. Nucleic Acids.

[65] Zhou Q., Liu L., Zhou J., Chen Y., Xie D., Yao Y., Cui D. (2021). Novel Insights Into MALAT1 Function as a MicroRNA Sponge in NSCLC. Front. Oncol..

[66] Chen W., Tan X., Yang Q., Fang Z., Xu Y. (2022). MALAT1 enhances gemcitabine resistance in non-small cell lung cancer cells by directly affecting miR-27a-5p/PBOV1 axis. Cell Signal.

[67] Roh J., Kim B., Im M., Jang W., Chae Y., Kang J., Youn B., Kim W. (2023). MALAT1-regulated gene expression profiling in lung cancer cell lines. BMC Cancer.

[68] Tiansheng G., Junming H., Xiaoyun W., Peixi C., Shaoshan D., Qianping C. (2020). lncRNA Metastasis-Associated Lung Adenocarcinoma Transcript 1 Promotes Proliferation and Invasion of Non-Small Cell Lung Cancer Cells via Down-Regulating miR-202 Expression. Cell J..

[69] Nazari M., Babakhanzadeh E., Mollazadeh A., Ahmadzade M., Mohammadi Soleimani E., Hajimaqsoudi E. (2024). HOTAIR in cancer: Diagnostic, prognostic, and therapeutic perspectives. Cancer Cell Int..

[70] Yao X., Wang T., Sun M.Y., Yuming Y., Guixin D., Liu J. (2022). Diagnostic value of lncRNA HOTAIR as a biomarker for detecting and staging of non-small cell lung cancer. Biomarkers.

[71] Li H.S., Xu Y. (2020). Inhibition of EZH2 via the STAT3/HOTAIR signalling axis contributes to cell cycle arrest and apoptosis induced by polyphyllin I in human non-small cell lung cancer cells. Steroids.

[72] Yu Y., Ren K. (2021). Five long non-coding RNAs establish a prognostic nomogram and construct a competing endogenous RNA network in the progression of non-small cell lung cancer. BMC Cancer.

[73] Chen D., Li Y., Wang Y., Xu J. (2021). LncRNA HOTAIRM1 knockdown inhibits cell glycolysis metabolism and tumor progression by miR-498/ABCE1 axis in non-small cell lung cancer. Genes Genom..

[74] Zhu C., Wang X., Wang Y., Wang K. (2022). Functions and underlying mechanisms of lncRNA HOTAIR in cancer chemotherapy resistance. Cell Death Discov..

[75] Zhang X., Luo M., Zhang J., Guo B., Singh S., Lin X., Xiong H., Ju S., Wang L., Zhou Y. (2022). The role of lncRNA H19 in tumorigenesis and drug resistance of human Cancers. Front. Genet..

[76] Zhang R., Zheng Y., Zhu Q., Gu X., Xiang B., Gu X., Xie T., Sui X. (2024). β-Elemene Reverses Gefitinib Resistance in NSCLC Cells by Inhibiting lncRNA H19-Mediated Autophagy. Pharmaceuticals.

[77] Lei Y., Guo W., Chen B., Chen L., Gong J., Li W. (2018). Tumor-released lncRNA H19 promotes gefitinib resistance via packaging into exosomes in non-small cell lung cancer. Oncol. Rep..

[78] Gu B., Yang M., Shi L., Yuan G., Xie H., Ni B. (2023). Progranulin modulates the progression of non-small cell lung cancer through lncRNA H19. Am. J. Transl. Res..

[79] Xia Y., Pei T., Zhao J., Wang Z., Shen Y., Yang Y., Liang J. (2024). Long noncoding RNA H19: Functions and mechanisms in regulating programmed cell death in cancer. Cell Death Discov..

[80] Liu L., Liu L., Lu S. (2019). lncRNA H19 promotes viability and epithelial-mesenchymal transition of lung adenocarcinoma cells by targeting miR-29b-3p and modifying STAT3. Int. J. Oncol..

[81] Tang F., Tian L.H., Zhu X.H., Yang S., Zeng H., Yang Y.Y. (2024). METTL3-mediated m6A modification enhances lncRNA H19 stability to promote endothelial cell inflammation and pyroptosis to aggravate atherosclerosis. Faseb J..

[82] Qi L., Yin Y., Sun M. (2023). m6A-mediated lncRNA NEAT1 plays an oncogenic role in non-small cell lung cancer by upregulating the HMGA1 expression through binding miR-361-3p. Genes Genom..

[83] Hussain M.S., Afzal O., Gupta G., Goyal A., Almalki W.H., Kazmi I., Alzarea S.I., Alfawaz Altamimi A.S., Kukreti N., Chakraborty A. (2024). Unraveling NEAT1’s complex role in lung cancer biology: A comprehensive review. EXCLI J..

[84] Zhang J., Li Y., Dong M., Wu D. (2017). Long non-coding RNA NEAT1 regulates E2F3 expression by competitively binding to miR-377 in non-small cell lung cancer. Oncol. Lett..

[85] Sun C., Li S., Zhang F., Xi Y., Wang L., Bi Y., Li D. (2016). Long non-coding RNA NEAT1 promotes non-small cell lung cancer progression through regulation of miR-377-3p-E2F3 pathway. Oncotarget.

[86] Zhao L., Bi M., Zhang H., Shi M. (2020). Downregulation of NEAT1 Suppresses Cell Proliferation, Migration, and Invasion in NSCLC Via Sponging miR-153-3p. Cancer Biother. Radiopharm..

[87] Zhao M.M., Ge L.Y., Yang L.F., Zheng H.X., Chen G., Wu L.Z., Shi S.M., Wang N., Hang Y.P. (2020). LncRNA NEAT1/miR-204/NUAK1 Axis is a Potential Therapeutic Target for Non-Small Cell Lung Cancer. Cancer Manag. Res..

[88] Wei C.M., Zhao X.F., Qiu H.B., Ming Z., Liu K., Yan J. (2020). The long non-coding RNA PVT1/miR-145-5p/ITGB8 axis regulates cell proliferation, apoptosis, migration and invasion in non-small cell lung cancer cells. Neoplasma.

[89] Zeng S.H.G., Xie J.H., Zeng Q.Y., Dai S.H.H., Wang Y., Wan X.M., Liu J.C.H. (2021). lncRNA PVT1 Promotes Metastasis of Non-Small Cell Lung Cancer Through EZH2-Mediated Activation of Hippo/NOTCH1 Signaling Pathways. Cell J..

[90] Li M.Y., Tang X.H., Fu Y., Wang T.J., Zhu J.M. (2019). Regulatory Mechanisms and Clinical Applications of the Long Non-coding RNA PVT1 in Cancer Treatment. Front. Oncol..

[91] Qin S., Zhao Y., Lim G., Lin H., Zhang X., Zhang X. (2019). Circular RNA PVT1 acts as a competing endogenous RNA for miR-497 in promoting non-small cell lung cancer progression. Biomed. Pharmacother..

[92] Ryter S.W., Cloonan S.M., Choi A.M. (2013). Autophagy: A critical regulator of cellular metabolism and homeostasis. Mol. Cells.

[93] Mizushima N., Komatsu M. (2011). Autophagy: Renovation of cells and tissues. Cell.

[94] White E. (2015). The role for autophagy in cancer. J. Clin. Investig..

[95] Rakesh R., PriyaDharshini L.C., Sakthivel K.M., Rasmi R.R. (2022). Role and regulation of autophagy in cancer. Biochim. Biophys. Acta Mol. Basis Dis..

[96] Liu G., Pei F., Yang F., Li L., Amin A.D., Liu S., Buchan J.R., Cho W.C. (2017). Role of Autophagy and Apoptosis in Non-Small-Cell Lung Cancer. Int. J. Mol. Sci..

[97] Chen S., Saeed A., Liu Q., Jiang Q., Xu H., Xiao G.G., Rao L., Duo Y. (2023). Macrophages in immunoregulation and therapeutics. Signal Transduct. Target. Ther..

[98] Klionsky D.J., Abdalla F.C., Abeliovich H., Abraham R.T., Acevedo-Arozena A., Adeli K., Agholme L., Agnello M., Agostinis P., Aguirre-Ghiso J.A. (2012). Guidelines for the use and interpretation of assays for monitoring autophagy. Autophagy.

[99] Amaravadi R., Kimmelman A.C., White E. (2016). Recent insights into the function of autophagy in cancer. Genes Dev..

[100] Parzych K.R., Klionsky D.J. (2014). An overview of autophagy: Morphology, mechanism, and regulation. Antioxid. Redox Signal.

[101] Yorimitsu T., Klionsky D.J. (2005). Autophagy: Molecular machinery for self-eating. Cell Death Differ..

[102] Ravikumar B., Sarkar S., Davies J.E., Futter M., Garcia-Arencibia M., Green-Thompson Z.W., Jimenez-Sanchez M., Korolchuk V.I., Lichtenberg M., Luo S. (2010). Regulation of mammalian autophagy in physiology and pathophysiology. Physiol. Rev..

[103] Huang J., Wang J. (2025). Selective protein degradation through chaperone-mediated autophagy: Implications for cellular homeostasis and disease (Review). Mol. Med. Rep..

[104] Kaushik S., Cuervo A.M. (2018). The coming of age of chaperone-mediated autophagy. Nat. Rev. Mol. Cell Biol..

[105] Yu X., Ye X., Lin H., Feng N., Gao S., Zhang X., Wang Y., Yu H., Deng X., Qian B. (2018). Knockdown of long non-coding RNA LCPAT1 inhibits autophagy in lung cancer. Cancer Biol. Med..

[106] Mizushima N., Levine B. (2010). Autophagy in mammalian development and differentiation. Nat. Cell Biol..

[107] Dikic I., Elazar Z. (2018). Mechanism and medical implications of mammalian autophagy. Nat. Rev. Mol. Cell Biol..

[108] Cai Q., Wang S., Jin L., Weng M., Zhou D., Wang J., Tang Z., Quan Z.J.M.c. (2019). Long non-coding RNA GBCDRlnc1 induces chemoresistance of gallbladder cancer cells by activating autophagy. Mol. Cancer.

[109] Feng Y., He D., Yao Z., Klionsky D.J. (2014). The machinery of macroautophagy. Cell Res..

[110] Jahangiri L., Ishola T., Pucci P., Trigg R.M., Pereira J., Williams J.A., Cavanagh M.L., Gkoutos G.V., Tsaprouni L., Turner S.D. (2021). The role of autophagy and lncRNAs in the maintenance of cancer stem cells. Cancers.

[111] Pustovalova M., Alhaddad L., Blokhina T., Smetanina N., Chigasova A., Chuprov-Netochin R., Eremin P., Gilmutdinova I., Osipov A.N., Leonov S. (2021). The CD44high subpopulation of multifraction irradiation-surviving NSCLC cells exhibits partial EMT-program activation and DNA damage response depending on their p53 status. Int. J. Mol. Sci..

[112] Li N., Gao W.J., Liu N.S. (2017). LncRNA BCAR4 promotes proliferation, invasion and metastasis of non-small cell lung cancer cells by affecting epithelial-mesenchymal transition. Eur. Rev. Med. Pharmacol. Sci..

[113] Huang L., Liu X., Chen Q., Yang J., Zhang D., Zhao Y., Xu L., Li Z., Liu X., Shao S. (2024). TGF-β-induced lncRNA TBUR1 promotes EMT and metastasis in lung adenocarcinoma via hnRNPC-mediated GRB2 mRNA stabilization. Cancer Lett..

[114] Qin P., Li Q., Zu Q., Dong R., Qi Y. (2024). Natural products targeting autophagy and apoptosis in NSCLC: A novel therapeutic strategy. Front. Oncol..

[115] Liu M., Fan Y., Li D., Han B., Meng Y., Chen F., Liu T., Song Z., Han Y., Huang L. (2021). Ferroptosis inducer erastin sensitizes NSCLC cells to celastrol through activation of the ROS–mitochondrial fission–mitophagy axis. Mol. Oncol..

[116] Ariosa A.R., Lahiri V., Lei Y., Yang Y., Yin Z., Zhang Z., Klionsky D.J. (2021). A perspective on the role of autophagy in cancer. Biochim. Biophys. Acta Mol. Basis Dis..

[117] Ma Q., Chen K., Xiao H. (2025). Rapamycin combined with osimertinib alleviated non-small cell lung cancer by regulating the PARP, Akt/mTOR, and MAPK/ERK signaling pathways. Front. Mol. Biosci..

[118] Ávalos Y., Canales J., Bravo-Sagua R., Criollo A., Lavandero S., Quest A.F. (2014). Tumor suppression and promotion by autophagy. Biomed. Res. Int..

[119] Stefanou D.T., Kouvela M., Stellas D., Voutetakis K., Papadodima O., Syrigos K., Souliotis V.L. (2022). Oxidative stress and deregulated DNA damage response network in lung cancer patients. Biomedicines.

[120] Carretero-Fernández M., Cabrera-Serrano A.J., Sánchez-Maldonado J.M., Ruiz-Durán L., Jiménez-Romera F., García-Verdejo F.J., González-Olmedo C., Cardús A., Díaz-Beltrán L., Gutiérrez-Bautista J.F. (2025). Autophagy and Oxidative Stress in Solid Tumors: Mechanisms and Therapeutic Opportunities. Crit. Rev. Oncol. Hematol..

[121] Kumar P., Choudhary A., Kinger S., Jagtap Y.A., Prajapati V.K., Chitkara D., Chinnathambi S., Verma R.K., Mishra A. (2025). Autophagy as a potential therapeutic target in regulating improper cellular proliferation. Front. Pharmacol..

[122] Chen X., He Q., Zeng S., Xu Z. (2022). Upregulation of nuclear division cycle 80 contributes to therapeutic resistance via the promotion of autophagy-related protein-7-dependent autophagy in lung cancer. Front. Pharmacol..

[123] Hu J., Zhang P.-J., Zhang D., Chen Z.-H., Cao X.-C., Yu Y., Ge J. (2022). An autophagy-associated lncRNAs model for predicting the survival in non-small cell lung cancer patients. Front. Genet..

[124] Liu T.-T., Li R., Liu X., Zhou X.-J., Huo C., Li J.-P., Qu Y.-Q. (2021). LncRNA XIST acts as a MicroRNA-520 sponge to regulate the Cisplatin resistance in NSCLC cells by mediating BAX through CeRNA network. Int. J. Med. Sci..

[125] Kumar A., Girisa S., Alqahtani M.S., Abbas M., Hegde M., Sethi G., Kunnumakkara A.B. (2023). Targeting Autophagy Using Long Non-Coding RNAs (LncRNAs): New Landscapes in the Arena of Cancer Therapeutics. Cells.

[126] Wang Y., Salai A., Luo D., Lv H., Gao S., Kamili A., Aishanjiang D., Liu Y. (2025). Construction of a prognostic model for autophagy-related LncRNAs in lung adenocarcinoma. Medicine.

[127] Yang D., Ma X., Song P. (2022). A prognostic model of non small cell lung cancer based on TCGA and ImmPort databases. Sci. Rep..

[128] Zhang C., Zhou Y., Zhang B., Sheng Z., Sun N., Yuan B., Wu X. (2023). Identification of lncRNA, miRNA and mRNA expression profiles and ceRNA Networks in small cell lung cancer. BMC Genom..

[129] Braga E.A., Fridman M.V., Burdennyy A.M., Loginov V.I., Dmitriev A.A., Pronina I.V., Morozov S.G. (2023). Various LncRNA Mechanisms in Gene Regulation Involving miRNAs or RNA-Binding Proteins in Non-Small-Cell Lung Cancer: Main Signaling Pathways and Networks. Int. J. Mol. Sci..

[130] Ding D., Zhang J., Luo Z., Wu H., Lin Z., Liang W., Xue X. (2022). Analysis of the lncRNA–miRNA–mRNA network reveals a potential regulatory mechanism of EGFR-TKI resistance in NSCLC. Front. Genet..

[131] Zhang Y., Tang J., Wang C., Zhang Q., Zeng A., Song L. (2024). Autophagy-related lncRNAs in tumor progression and drug resistance: A double-edged sword. Genes Dis..

[132] Jiao J., Zhao Y., Li Q., Jin S., Liu Z. (2024). LncRNAs in tumor metabolic reprogramming and tumor microenvironment remodeling. Front. Immunol..

[133] Li C., Liu J.-H., Su J., Lin W.-J., Zhao J.-Q., Zhang Z.-H., Wu Q. (2021). LncRNA XIST knockdown alleviates LPS-induced acute lung injury by inactivation of XIST/miR-132-3p/MAPK14 pathway: XIST promotes ALI via miR-132-3p/MAPK14 axis. Mol. Cell. Biochem..

[134] Huang F.X., Chen H.J., Zheng F.X., Gao Z.Y., Sun P.F., Peng Q., Liu Y., Deng X., Huang Y.H., Zhao C. (2019). LncRNA BLACAT1 is involved in chemoresistance of non-small cell lung cancer cells by regulating autophagy. Int. J. Oncol..

[135] Borzi C., Ganzinelli M., Caiola E., Colombo M., Centonze G., Boeri M., Signorelli D., Caleca L., Rulli E., Busico A. (2021). LKB1 Down-Modulation by miR-17 Identifies Patients With NSCLC Having Worse Prognosis Eligible for Energy-Stress–Based Treatments. J. Thorac. Oncol..

[136] Li J., Gan J., Shi S., Huang J., Yang Y. (2025). The potential of targeting autophagy-related non-coding RNAs in the treatment of lung cancer. Front. Pharmacol..

[137] Luong T.V., Cao M.T.T., Nguyen N.V.D., Dang H.N.N., Nguyen T.T. (2025). Roles of autophagy and long non-coding RNAs in gastric cancer. World J. Gastroenterol..

[138] Xu L., Huang X., Lou Y., Xie W., Zhao H. (2022). Regulation of apoptosis, autophagy and ferroptosis by non-coding RNAs in metastatic non-small cell lung cancer. Exp. Ther. Med..

[139] Zheng T., Li D., He Z., Feng S., Zhao S. (2018). Long noncoding RNA NBAT1 inhibits autophagy via suppression of ATG7 in non-small cell lung cancer. Am. J. Cancer Res..

[140] Sun W., Zu Y., Fu X., Deng Y.J.O.r. (2017). Knockdown of lncRNA-XIST enhances the chemosensitivity of NSCLC cells via suppression of autophagy. Oncol. Rep..

[141] Giordo R., Ahmadi F.A.M., Husaini N.A., Al-Nuaimi N., Ahmad S.M.S., Pintus G., Zayed H. (2024). microRNA 21 and long non-coding RNAs interplays underlie cancer pathophysiology: A narrative review. Non-Coding RNA Res..

[142] Rama Ballesteros A.R., Quiñonero Muñoz F.J., Mesas Hernández C., Melguizo Alonso C., Prados Salazar J.C. (2022). Synthetic Circular miR-21 Sponge as Tool for Lung Cancer Treatment. Int. J. Mol. Sci..

[143] Li Y., Liang Z., He H., Huang X., Mo Z., Tan J., Guo W., Zhao Z., Wei S. (2021). The lncRNA HOTAIR regulates autophagy and affects lipopolysaccharide-induced acute lung injury through the miR-17-5p/ATG2/ATG7/ATG16 axis. J. Cell Mol. Med..

[144] Zheng Y., Jia H., Wang P., Liu L., Chen Z., Xing X., Wang J., Tan X., Wang C. (2023). Silencing TRAIP suppresses cell proliferation and migration/invasion of triple negative breast cancer via RB-E2F signaling and EMT. Cancer Gene Ther..

[145] Shen W., Hong X., Jin C., Xi Y. (2021). LncRNA PSMG3AS1 promotes proliferation of non-small cell lung cancer cells by sponging miR-613 to upregulate SphK1. Cell Cycle.

[146] Liu X., Zhang X., Du S. (2022). Long non-coding RNA ACTA2-AS1 inhibits the cisplatin resistance of non-small cell lung cancer cells through inhibiting autophagy by suppressing TSC2. Cell Cycle.

[147] Vessoni A.T., Filippi-Chiela E.C., Menck C.F., Lenz G. (2013). Autophagy and genomic integrity. Cell Death Differ..

[148] Gadhave D.G., Sugandhi V.V., Jha S.K., Nangare S.N., Gupta G., Singh S.K., Dua K., Cho H., Hansbro P.M., Paudel K.R. (2024). Neurodegenerative disorders: Mechanisms of degeneration and therapeutic approaches with their clinical relevance. Ageing Res. Rev..

[149] Chen Z.H., Cao J.F., Zhou J.S., Liu H., Che L.Q., Mizumura K., Li W., Choi A.M., Shen H.H. (2014). Interaction of caveolin-1 with ATG12-ATG5 system suppresses autophagy in lung epithelial cells. Am. J. Physiol. Lung Cell Mol. Physiol..

[150] Nakano Y., Isobe K., Yoshizawa T., Urabe N., Homma S., Kishi K. (2023). Upregulation of long non-coding RNA LINC00460 in EGFR-mutant lung cancer indicates a poor prognosis in patients treated with osimertinib. Oncol. Lett..

[151] Jin K.T., Lu Z.B., Lv J.Q., Zhang J.G. (2020). The role of long non-coding RNAs in mediating chemoresistance by modulating autophagy in cancer. RNA Biol..

[152] Zhang H., Yang X., Guo Y., Zhao H., Jiang P., Yu Q.Q. (2025). The regulatory role of lncRNA in tumor drug resistance: Refracting light through a narrow aperture. Oncol. Res..

[153] Dong Y., He Y., Geng Y., Wei M., Zhou X., Lian J., Hallajzadeh J. (2024). Autophagy-related lncRNAs and exosomal lncRNAs in colorectal cancer: Focusing on lncRNA-targeted strategies. Cancer Cell Int..

[154] Lei X., Zheng Y., Su W. (2025). RNA-binding proteins and autophagy in lung cancer: Mechanistic insights and therapeutic perspectives. Discov. Oncol..

[155] Liu Z.Y., Tang J.M., Yang M.Q., Yang Z.H., Xia J.Z. (2024). The role of LncRNA-mediated autophagy in cancer progression. Front. Cell Dev. Biol..

[156] She K., He S., Lu X., Yu S., Li M., Xiong W., Zhou M. (2023). LncRNA SNHG7 promotes non-small cell lung cancer progression and cisplatin resistance by inducing autophagic activity. J. Thorac. Dis..

[157] Sun Y., Jin S.D., Zhu Q., Han L., Feng J., Lu X.Y., Wang W., Wang F., Guo R.H. (2017). Long non-coding RNA LUCAT1 is associated with poor prognosis in human non-small lung cancer and regulates cell proliferation via epigenetically repressing p21 and p57 expression. Oncotarget.

[158] Liu X., Zhou X., Chen Y., Huang Y., He J., Luo H. (2019). miR-186-5p targeting SIX1 inhibits cisplatin resistance in non-small-cell lung cancer cells (NSCLCs). Neoplasma.

[159] Xi S., Ming D.J., Zhang J.H., Guo M.M., Wang S.Y., Cai Y., Liu M.Y., Wang D.Q., Zhang Y.J., Li Y. (2023). Downregulation of N6-methyladenosine-modified LINC00641 promotes EMT, but provides a ferroptotic vulnerability in lung cancer. Cell Death Dis..

[160] Mu D., Han B., Huang H., Zheng Y., Zhang J., Shi Y. (2025). Unraveling the advances of non-coding RNAs on the tumor microenvironment: Innovative strategies for cancer therapies. J. Transl. Med..

[161] Du Y., Zhu S., Liu X., Sun Y., Cui T., Liu J., Zhang W., Shao S. (2025). LncRNA HOTAIR regulates the expression of MRP1 gene through the mir-6807-5p/Egr1 axis to affect the multidrug resistance of lung cancer cells. Gene.

[162] You H., Wang L., Meng H., Li J., Fang G. (2025). Autophagy: Shedding Light on the Mechanisms and Multifaceted Roles in Cancers. Biomolecules.

[163] Chen B., Dragomir M.P., Yang C., Li Q., Horst D., Calin G.A. (2022). Targeting non-coding RNAs to overcome cancer therapy resistance. Signal Transduct. Target. Ther..

[164] Liu Z., Liu X., Yin C., Liu Z., Yu H. (2025). Identification of circRNA-Based Biomarkers and ceRNA Mechanism in Non-Small Cell Lung Cancer. Cell Biochem. Biophys..

[165] Ju Z.S., Sun B., Bao D., Zhang X.F. (2020). Effect of lncRNA-BLACAT1 on drug resistance of non-small cell lung cancer cells in DDP chemotherapy by regulating cyclin D1 expression. Eur. Rev. Med. Pharmacol. Sci..

[166] Xu X., Zhou X., Chen Z., Gao C., Zhao L., Cui Y. (2020). Silencing of lncRNA XIST inhibits non-small cell lung cancer growth and promotes chemosensitivity to cisplatin. Aging.

[167] Yang Y., Jiang C., Yang Y., Guo L., Huang J., Liu X., Wu C., Zou J. (2018). Silencing of LncRNA-HOTAIR decreases drug resistance of Non-Small Cell Lung Cancer cells by inactivating autophagy via suppressing the phosphorylation of ULK1. Biochem. Biophys. Res. Commun..

[168] Ke C., Feng X., Li J., Chen S., Hu X. (2022). Association between long non-coding RNA HOTAIR polymorphism and lung cancer risk: A systematic review and meta-analysis. Exp. Ther. Med..

[169] Suri C., Swarnkar S., Bhaskar L., Verma H.K. (2024). Non-Coding RNA as a Biomarker in Lung Cancer. Non-Coding RNA.

[170] Rasul M.F., Hussen B.M., Salihi A., Ismael B.S., Jalal P.J., Zanichelli A., Jamali E., Baniahmad A., Ghafouri-Fard S., Basiri A. (2022). Strategies to overcome the main challenges of the use of CRISPR/Cas9 as a replacement for cancer therapy. Mol. Cancer.

